# Spontaneous rupture of the pancreatic arcade artery caused by neurofibromatosis type 1 successfully treated using emergency transcatheter arterial embolization, partial intra-aortic balloon occlusion, and stent graft placement: a case report and review of the literature

**DOI:** 10.1186/s42155-020-00129-y

**Published:** 2020-07-26

**Authors:** Ryo Morita, Daisuke Abo, Takeshi Soyama, Yuki Yoshino, Toru Yoshikawa, Tasuku Kimura, Kohsuke Kudo

**Affiliations:** 1 Department of Diagnostic and Interventional Radiology, Hokkaido University Hospital, Sapporo, Japan; 2 Department of Diagnostic Radiology, National Hospital Organization Hokkaido Cancer Center, Sapporo, Japan; 3 Department of Diagnostic Imaging, Hokkaido University Graduate School Medicine, Hokkaido University, Sapporo, Japan

**Keywords:** Neurofibromatosis type 1, Stent-graft placement, Endovascular therapy, Pancreatic arcade artery, Spontaneous rupture, Vasculopathy, Intra-aortic balloon occlusion

## Abstract

**Background:**

Vascular abnormalities in neurofibromatosis type 1 (NF1) are rare, but are the second leading cause of death in persons with NF1. In NF1 vasculopathy (NF-V), fatal bleeding due to a spontaneous arterial rupture sometimes occurs. Ruptured extracranial arteries in patients with NF1 often involve thoracic vessels, such as the intercostal and subclavian arteries; very few reports exist regarding the abdominal region. Herein, we present the first case of intraperitoneal bleeding due to spontaneous pancreatic arcade artery (PAA) rupture associated with NF1, successfully treated by transcatheter arterial embolization (TAE) combined with stent-graft placement and partial intra-aortic balloon occlusion (IABO).

**Case presentation:**

A 40-year-old woman complained of back and abdominal pain. Upon admission, her blood pressure was 85/41 mmHg and heart rate was 129 beats/min. Computed tomography (CT) showed large intraperitoneal bleeding due to PAA rupture. After CT scanning, her systolic blood pressure decreased to 50 mmHg. Therefore, we performed emergency TAE with partial IABO. She was treated by TAE of the anterior superior pancreaticoduodenal artery, anterior inferior pancreaticoduodenal artery, and inferior pancreaticoduodenal artery. However, even after TAE, minor extravasation around the superior mesenteric artery continued, and her vital signs remained unstable. Stent-graft placement was selected to stop the haemorrhage, preserving normal blood flow of the superior mesenteric artery trunk. Excellent patency of the stent graft was confirmed on follow-up CT, and she was discharged on postoperative day 56.

**Conclusion:**

PAA rupture associated with NF1 can be successfully treated by TAE combined with partial intra-aortic balloon occlusion, and stent-graft placement.

## Background

Vascular abnormalities in neurofibromatosis type 1 (NF1) are rare, with an incidence of 0.4–6.4% (Raborn et al. 2020); however, fatal bleeding can occur due to a spontaneous arterial rupture. NF1 vasculopathy (NF-V) is the second most common cause of mortality (Rasmussen et al. 2001). Among 19 cases of ruptured extracranial arteries in NF1 reported from 1996 to 2016 (Huffman et al. 1996; Ishizu et al. 2006; Hinsch et al. 2008; Aizawa et al. 2010; Falcone et al. 2010; Hung et al. 2012; Park et al. 2012; Moerbeek et al. 2015; Liang et al. 2016), the most common bleeding sites comprised thoracic vessels, such as the intercostal, subclavian, and internal thoracic arteries. Only two ruptures of visceral arteries (superior mesenteric artery (SMA) trunk and renal artery) have been reported (Huffman et al. 1996; Hinsch et al. 2008). As yet, none have involved a spontaneous rupture of the pancreatic arcade artery (PAA). Herein, we present the first case of intraperitoneal bleeding due to spontaneous PAA rupture associated with NF1, successfully treated by transcatheter arterial embolization (TAE) combined with stent-graft placement under partial intra-aortic balloon occlusion (IABO).

## Case presentation

**Fig. 1:**
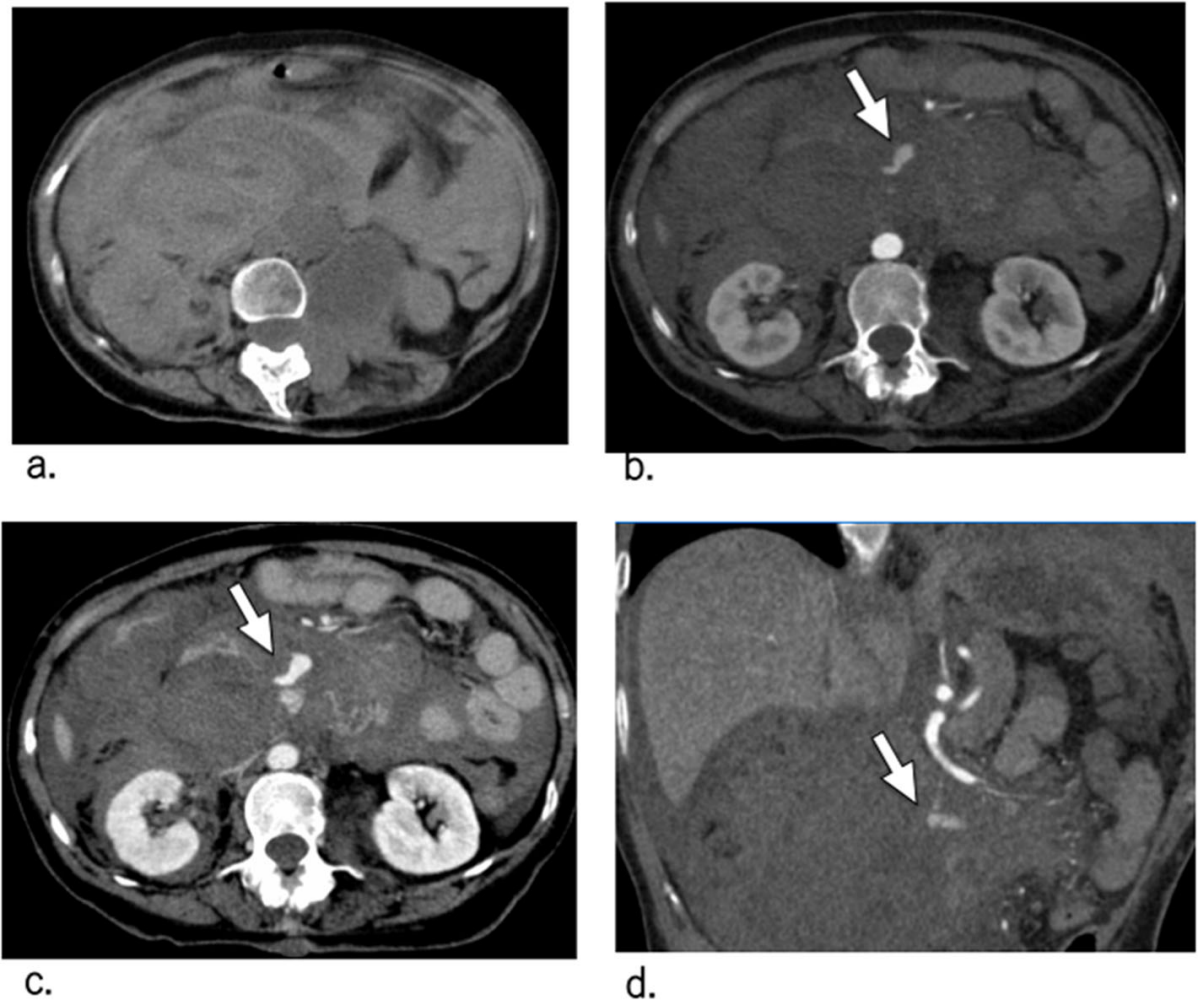
Computed tomography (CT) at symptom presentation. **a** Non-contrast-enhanced CT image showing a large retroperitoneal hematoma around the duodenum and haemorrhagic ascites. **b** Contrast-enhanced CT arterial phase image showing a large retroperitoneal hematoma with definite pseudoaneurysm (white arrow). **c** Contrast-enhanced CT portal venous phase image showing a large retroperitoneal hematoma with definite pseudoaneurysm (white arrow). **d** Contrast-enhanced CT arterial phase coronal images (slab-maximum intensity projection) showing the superior mesenteric artery (SMA) and pseudoaneurysm

A 40-year old woman, diagnosed with NF1 complained of acute, severe back and abdominal pain during work. She was transferred to the emergency room at our hospital due to syncope. Upon admission, her blood pressure was 85/41 mmHg and heart rate was 129 beats/min. Computed tomography (CT) showed a large retroperitoneal hematoma, with extravasation around the duodenum, and intraperitoneal blood (Fig. 1a,b). After CT scanning, her systolic blood pressure decreased to 50 mmHg. Laboratory data revealed severe anaemia (haemoglobin, 5.4 mg/mL) and mild coagulopathy (prothrombin time-international normalized ratio, 1.31; activated partial thromboplastin time, 41.0 s). No thrombocytopenia (platelets, 225 × 1000/μL) and no history of anticoagulant therapy were noted. Her blood pressure was only slightly increased to 70 mmHg by a rapid infusion of packed red blood cells (two units); thus, an intra-aortic balloon (Rescue Balloon®-ER, Tokai Medical Products, Inc., Aichi, Japan) was introduced via the left femoral artery, and balloon was partially inflated at the lower level of the thoracic aorta to stabilize her systemic blood pressure. Because we suspected a PAA rupture associated with NF1, based on CT findings (Fig. 1c,d), emergency TAE of the PAA was planned shortly thereafter.

**Fig. 2:**
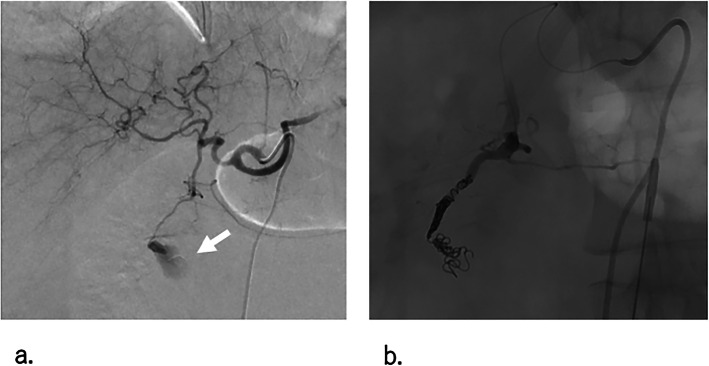
Celiac artery angiography at emergency transarterial embolization (TAE). **a** Celiac artery angiogram showing extravasation (white arrow) from the ASPDA, and its disruption. In addition, no median ligament compression syndrome was noted. **b** Digital angiogram, obtained after TAE of the ASPDA, showing a pseudoaneurysm, and the disappearance of extravasation from the celiac artery system. The ASPDA appears to be dilated, because a severe vascular spasm due to haemorrhage was relieved after TAE. ※ ASPDA: anterior superior pancreaticoduodenal artery

After the introduction of a 5-F sheath via the right femoral artery, celiac arteriography was firstly performed, which showed extravasation from the anterior superior pancreaticoduodenal artery (ASPDA), as well as its disruption (Fig. 2a).

**Fig. 3:**
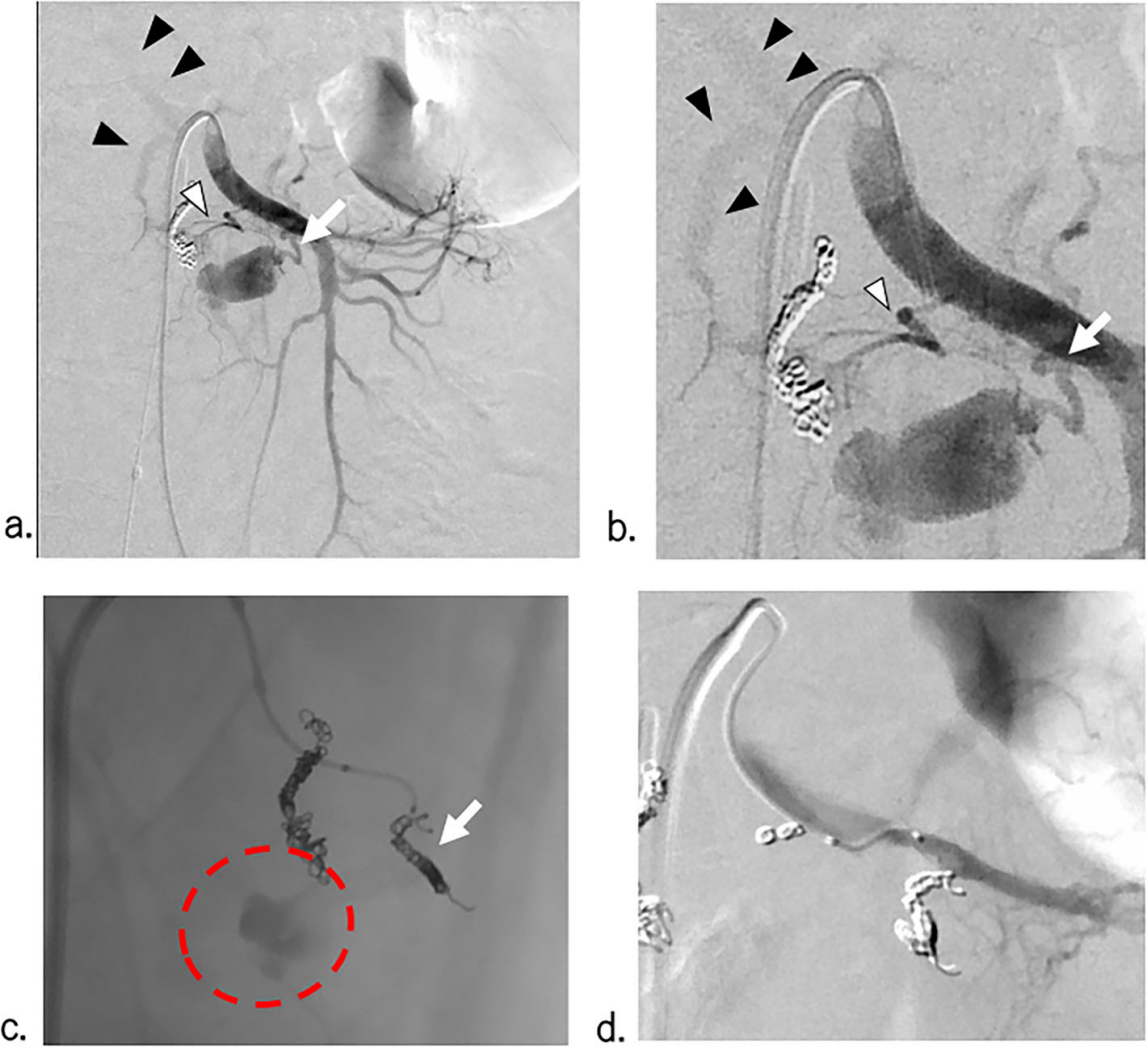
SMA angiography at emergency transarterial embolization (**a**) (**b**) SMA angiogram showing a rupture of the AIPDA (white arrow) and early visualization of the portal vein (black arrowheads). The PIPDA (white arrows) was suspected to be involved in the haemorrhage. **c** Digital angiogram, obtained after coil embolization of the proximal AIPDA (white arrow), showing continued extravasation (red circle) from the AIPDA. **d** AIPDA angiogram after 50% NBCA lipiodol injection to the AIPDA, showing the disappearance of extravasation, with maintained normal blood flow in the branch of the SMA near the AIPDA. ※ AIPDA: anterior inferior pancreaticoduodenal artery ※ PIPDA: posterior inferior pancreaticoduodenal artery. ※ NBCA: n-butyl-2 cyanoacrylate. ※ SMA: superior mesenteric artery

Because isolation of the pseudoaneurysm was difficult due to the disruption of the ASPDA, the ASPDA was embolized from the disrupted site to a healthy segment with five pushable platinum coils (2.0 mm diameter with 60 mm length, C-stopper coil, Piolax Medical Devices Inc., Kanagawa, Japan), using a 1.7-F coaxial microcatheter (ASAHI Veloute, Asahi Intecc Co., Aichi, Japan) advanced through a 4-F Mikaelson catheter (Fig. 2b). Aiming to isolate the disrupted PAA completely, angiography of the SMA was subsequently performed, which showed a huge pseudoaneurysm and extravasation of the anterior inferior pancreaticoduodenal artery (AIPDA), as expected. In addition, rupture of the posterior inferior pancreaticoduodenal artery (PIPDA) was also suspected, but was not definite (Fig. 3a). At this time, her blood pressure was still unstable. The catheter and sheath were changed to a 5.2-Fr balloon catheter (Selecon MP® balloon, TERUMO, Tokyo, Japan) and J-shaped sheath (Medikit, Tokyo, Japan) for temporal selective occlusion of the SMA (to stabilize the bleeding from the SMA), rather than a complete IABO. Selective catheterization via a microcatheter advanced through an inflated 5.2-Fr balloon catheter was difficult due to the steep angles of the PAA (both the AIPDA and PIPDA); thus, after several attempts, the microcatheter was changed to a 2.4-F steerable microcatheter (SwiftNINJA, Sumitomo Bakelite, Tokyo, Japan). A huge pseudoaneurysm of the AIPDA with early visualization of the portal vein, indicating an arterioportal fistula (Fig. 3b), was embolized with three detachable microcoils (2.0 mm diameter with 60 mm length, Target® Detachable Coils; Stryker Corporation, Kalamazoo, Michigan 49,002, USA) from the disrupted site to a healthy segment, as with the ASPDA. However, complete haemostasis was not obtained by coil embolization alone (Fig. 3c). Therefore, we added a 1:1 mixture of n-butyl-2 cyanoacrylate (NBCA; Histoacryl Blue; B. Braun, Melsungen, Germany) and iodized oil (Lipiodol; Guerbet, Aulnay- Sous-Bois, France). Complete flow stasis was achieved and the disrupted anterior PAA was isolated successfully. There was no overflow of glue into the SMA (Fig. 3d). The PIPDA was embolized by gelatin sponge particles, followed by two detachable microcoils (1.5 mm diameter with 30 mm length), as selective angiography of the PIPDA showed multiple minor bleeding sites via tiny its branches.

**Fig. 4:**
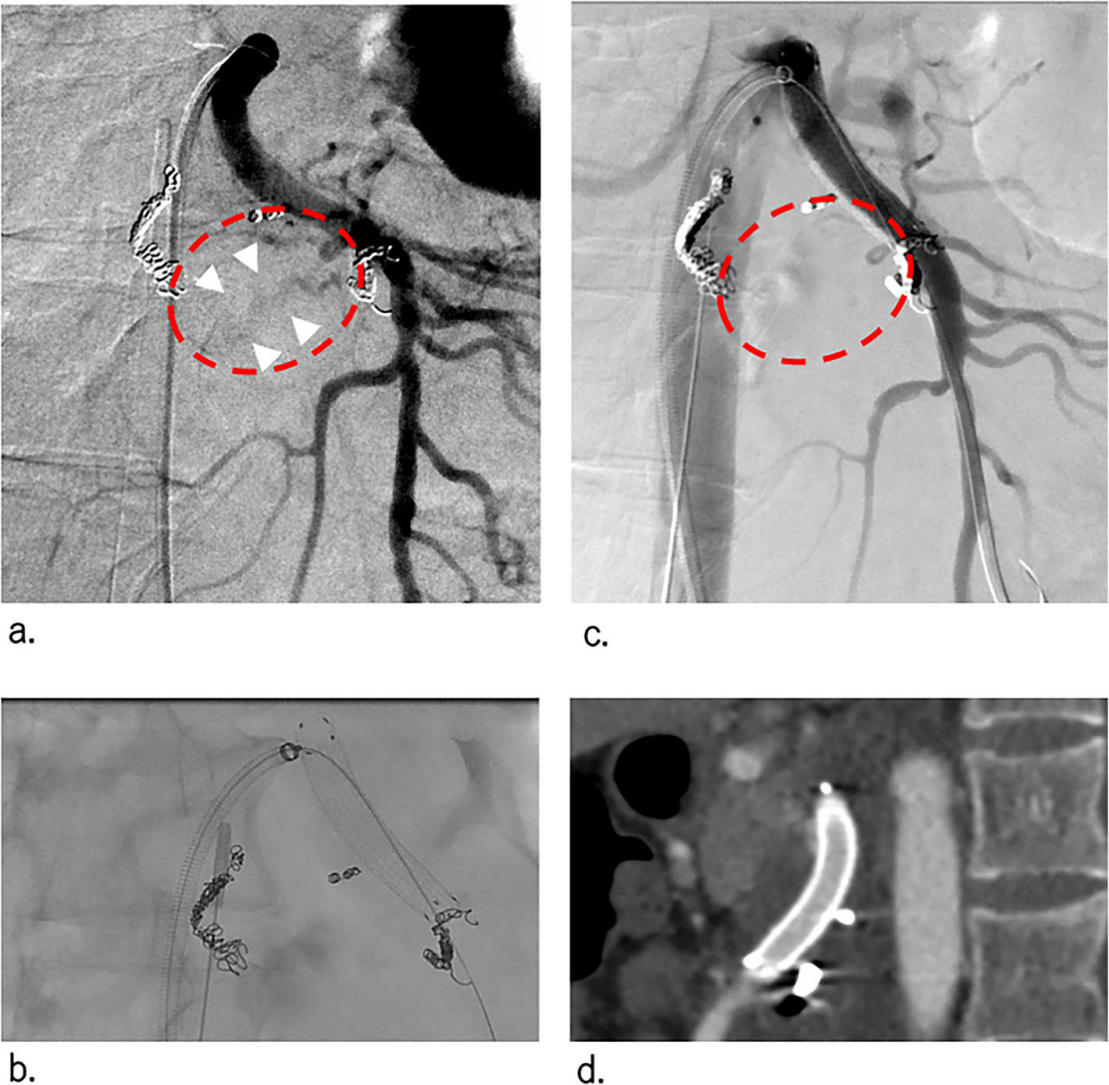
SMA angiography at stent-graft placement and postoperative computed tomography (CT) (**a**) SMA angiogram, obtained after TAE, showing continued extravasation (white arrowhead in the red circle) without a definite causal artery. **b** Digital angiogram obtained after stent-graft placement in the SMA trunk (Viabahn, 7 mm × 5 cm). **c** SMA angiogram, obtained after stent-graft placement, showing the complete disappearance of extravasation (red circle). **d** Contrast-enhanced CT image, obtained at day 28 after treatment, confirming good patency of the stent graft

However, even after complete TAE of the PAA, minor extravasation around the main trunk of the proximal SMA continued, and her vital signs remained unstable (Fig. 4a). Because bleeding from the main trunk of the SMA or a fine vessel was suspected (radiologically not confirmed), stent-graft placement (Viabahn® 7 mm × 5 cm; Gore & Associates, Inc., Flagstaff, AZ, USA) was selected to stop the haemorrhage and maintain the normal blood flow of the SMA trunk (Fig. 4b,c). Placement was performed using a 7-Fr guiding sheath (Flexor Tuohy-Borst Side Arm Introducer, 55 cm, Cook, Bloomington, IN, USA) and a stiff wire (V18, Boston Scientific, Marlborough, MA, USA). Extravasation completely disappeared immediately after stent-graft placement. Subsequently, her vital signs became stable.

Administration of aspirin (100 mg/day) and edoxaban tosilate hydrate (because of the huge plexiform neurofibromas; Lixiana®, Daiichi-Sankyo Co. Ltd., Tokyo, Japan, 30 mg/day) was initiated at 24 days after stent-graft placement, which was changed to aspirin (100 mg/day) and warfarin potassium (3 mg/day) at 31 days after treatment. Excellent patency of the stent graft was confirmed on follow-up contrast CT at 28 days after treatment (Fig. 4d). She was discharged at 56 days after treatment.

## Discussion and conclusion

NF1 is one of the most frequent autosomal-dominant inherited disorders, with an incidence of 1 in 3000 births (Falcone et al. 2010). There is a great variety of manifestations in NF1, involving mesenchymal tissue in musculoskeletal and cardiovascular systems (Hinsch et al. 2008). Although the estimated prevalence of NF-V has been reported as 0.4–6.4%, the actual frequency is unknown, as most patients with NF-V are asymptomatic throughout life. According to an autopsy series of patients with NF1 who died of other causes, vasculopathy was reported in 44% of cases (Salyer and Salyer 1974). Therefore, with the inclusion of asymptomatic NF-V, the frequency of vasculopathy in patients with NF1 may be higher than previously reported.

Patients with NF1 have a decreased life expectancy, by ∼15 years, compared to that of the general population. Malignant neoplasms are the most common cause of death in NF1, followed by vasculopathy (Rasmussen et al. 2001). Cardiovascular disease, haemorrhage, and embolism associated with vasculopathy are frequent causes of death in adult patients with NF1 (Zoller et al. 1995). NF-V includes aneurysms, aneurysmal dilatation, stenosis, rupture, and arteriovenous malformations (D’Errico et al. 2018; Hinsch et al. 2008; Falcone et al. 2010). Among these, aneurysmal dilatation is the most common form of vasculopathy (D’Errico et al. 2018). Additionally, the involvement of multiple vessels is a common characteristic of NF-V (Friedman et al. 2002). In fact, in the present case, multiple aneurysmal dilatations and multiple splenic aneurysms were observed on CT.

Fatal haemorrhaging due to the spontaneous rupture of extracranial arteries in NF1 has been reported, with thoracic vessels in most cases (Ishizu et al. 2006; Bargiela et al. 2018). In contrast, there are only 5 reported cases of ruptured visceral arteries, with SMA, renal artery, and gastroduodenal artery involvement (Huffman et al. 1996; Hinsch et al. 2008; Bargiela et al. 2018); none involved the spontaneous rupture of the PAA.

NF-V is usually described as mesodermal dysplasia or fibromuscular hyperplasia (Hinsch et al. 2008) (Raborn et al. 2020). Mutations in the NF1 gene result in decreased neurofibromin, with subsequent proliferation of endothelial and smooth muscle cells in the arteries and veins (Hung et al. 2012). As a result, the vascular tissue is fragile in patients with NF1 (Aizawa et al. 2010). Moreover, the following have been reported as causes of rupture in patients with NF1: 1) the direct invasion of neurofibromas; 2) compression of the vasa vasorum by neurofibromas; 3) physical movement (e.g. orthopaedic traction); and 4) pregnancy (Ishizu et al. 2006; Leier et al. 1972).

In the present case, in addition to the aforementioned fragile nature of the arterial vessel, we speculated that the spontaneous rupture of the PAA, rather than an existing aneurysm associated with NF1, might have resulted from a direct invasion of neurofibromas. The pseudoaneurysm involved multiple arteries (ASPDA, AIPDA, and PIPDA), and selective angiography of the AIPDA showed early visualization of the portal vein at the AIPDA. We believe that a minor arterioportal shunt was created when the pseudoaneurysm ruptured, and an arteriovenous malformation did not exist. These findings suggest a sudden rupture due to a direct invasion of neurofibromas, rather than the rupture of an existing aneurysm. In fact, a previous CT scan (performed 7 years before TAE at another hospital; images not shown) showed a soft-tissue density around the SMA and no aneurysm in the PAA. Additionally, coronal short-TI inversion recovery magnetic resonance imaging (MRI) performed before TAE (as follow-up for a meningocele from the lumbar spine to the sacrum at another hospital; images not shown), showed a high-intensity area around the SMA, indicating the presence of neurofibromas. We believe that these findings support our speculation. Additionally, although the possibility that an existing PAA aneurysm had ruptured cannot be completely ruled out, this was considered unlikely, as there was no median arcuate ligament stenosis on celiac angiography and no aneurysm on previous CT images. Even if the present case involved a ruptured PAA aneurysm, it is still of interest, as there are only 2 reported cases of ruptured pancreaticoduodenal artery aneurysm associated with NF1 (Serleth et al. 1998; Fukushima et al. 2020).

To our best knowledge, the present case is first reported case of intraperitoneal bleeding due to spontaneous PAA rupture associated with NF1, successfully treated by TAE combined with stent-graft placement and IABO. Endovascular management is safe and effective at all ages, even in haemodynamically unstable patients with NF1 (Bargiela et al. 2018). Vital signs in the present case were unstable; therefore, we used IABO, which is effective in non-traumatic, as well as traumatic, intra-abdominal haemorrhage (Hoehn et al. 2019). However, her vital signs remained unstable under IABO after ASPDA embolization. Therefore, we controlled the flow of the SMA at the area of major extravasation with a compliant balloon. After this, her vital signs became stable, and endovascular treatment of the SMA system (AIPDA and PIPDA) could be performed.

In cases of neurofibromatosis-associated aneurysms, endovascular treatment with coiling is the most commonly used technique (55/66 cases; 83.3%), followed by stent-graft placement (10/66 cases; 15.2%) (Bargiela et al. 2018). Furthermore, in half of the latter cases, as well as in the present case, coil embolization was performed in addition to stent-graft placement. Thus, this treatment method should be considered in cases of rupture. We used a steerable microcatheter due to the steep angles of both the AIPDA and PIPDA, resulting in a successful catheterization. This device, which has a remote-controlled flexible tip, manipulated using a dial in the handgrip, is effective in the quick catheterization of such challenging vessel cases (Soyama et al. 2017).

Surgical control of haemorrhages in NF1 is reportedly difficult, due to the high fragility of the involved vessels with this disease (Hung et al. 2012; Aizawa et al. 2010). Therefore, if an arterial rupture is suspected, endovascular therapy should be considered (Hung et al. 2012).

Currently, there are no standard recommendations for routine follow up in NF-V. Vascular complications, including spontaneous rupture, can occur over time (Hung et al. 2012). A spontaneous arterial rupture is rare, but can sometimes be fatal (Roberts et al. 2019). Therefore, careful observation with imaging is necessary (Bargiela et al. 2018). In the present case, we planned to perform regular CT examinations once a year. Additionally, as she underwent stent-graft placement, there is a risk of SMA thrombotic occlusion; thus, lifelong antiplatelet therapy with aspirin (100 mg/day) is necessary. Good long-term stent patency was reported in 9 of 11 cases (mean follow-up, 28 months), including 1 case with stent placement in the SMA and antiplatelet therapy (100 mg/day aspirin) (Kunzle et al. 2013).

Based on the present case, PAA rupture should be considered as one of the potential conditions of NF-V. Furthermore, spontaneous PAA rupture associated with NF1 can be successfully treated by TAE combined with stent-graft placement and partial IABO.

## Data Availability

Not applicable.

## Abbreviations

AIPDA: Anterior inferior pancreaticoduodenal artery
ASPDA: Anterior superior pancreaticoduodenal artery
CT: Computed tomography
IABO: Intra-aortic balloon occlusion
MRI: Magnetic resonance imaging
NF1: Neurofibromatosis type 1
NF-V: NF1 vasculopathy
PAA: Pancreatic arcade artery
PIPDA: Posterior inferior pancreaticoduodenal artery
SMA: Superior mesenteric artery
TAE: Transcatheter arterial embolization
